# Genome-wide Long Non-coding RNA Analysis Identified Circulating LncRNAs as Novel Non-invasive Diagnostic Biomarkers for Gynecological Disease

**DOI:** 10.1038/srep23343

**Published:** 2016-03-18

**Authors:** Wen-Tao Wang, Yu-Meng Sun, Wei Huang, Bo He, Ya-Nan Zhao, Yue-Qin Chen

**Affiliations:** 1 Key Laboratory of Gene Engineering of the Ministry of Education, State Key Laboratory for Biocontrol, School of Life Science, Sun Yat-sen University, Guangzhou 510275, China; 2 Dept of Obst & Gyn, Sun Yat-sen Memorial Hospital, Sun Yat-sen University, Guangzhou 510120, China

## Abstract

Increasing evidence indicates that long non-coding RNAs (lncRNAs) play important roles in human diseases. This study aimed to investigate the tissue and serum lncRNAs that are differentially expressed between patients with endometriosis, a gynecological disease, to evaluate the potential of these lncRNAs as non-invasive markers for the disease. The differentially expressed lncRNAs as competing endogenous RNAs (ceRNAs) were also analyzed to predict their functions in disease development. Genome-wide profiling of lncRNA expression patterns revealed that many lncRNAs were abnormally expressed between sera and tissuesof the patient samples. A set of aberrant differentially expressed lncRNAs were further validated in a validation cohort of 110 serum and 24 tissue samples. Functional analysis predicted that differentially expressed lncRNAs may participate in disease development through crosstalk between the ceRNAs of miRNAs and may be involved in a range of cellular pathways including steroid or hormone responses. We also found a unique set of lncRNAs that were associated with disease severity and progression, and their diagnostic values were also investigated. Our study demonstrated that lncRNAs could potentially serve as non-invasive biomarkers for the diagnosis of endometriosis and as important regulators in the progression of this disease.

Genome-wide human transcriptional studies have revealed a large number of non-protein-coding RNAs (ncRNAs), including short and long non-coding RNAs^1^,^2^. Emerging evidence has shown that long non-coding RNAs (lncRNAs), a less characterized class of molecules greater than 200 nucleotides (nt) in length, play important roles in a wide range of biological processes. LncRNAs, which are mRNA-like transcripts, are mainly transcribed by RNA polymerase II (RNA PII) and are polyadenylated, spliced, and primarily localized in the nucleus^3^,^4^. LncRNAs often form highly stable secondary structures, making it possible to quantitatively detect free RNAs in body fluids, such as serum^5^,^6^. These characteristics suggested that lncRNAs might not only be potential biomarkers for clinical diagnosis of the disease but also be vital factors in disease development. In recent years, reports have suggested that circulating lncRNAs exhibit a predictive value to serve as diagnostic biomarkers in prostate cancer^7^, gastric cancer^8^, B-cell neoplasms^9^, prenatal testing^10^,and heart failure^11^. More importantly, studies have demonstrated that dysregulated expression of lncRNAs can lead to the occurrence and progression of a number types of diseases, including cancer^12^, leukemia^13^, and diabetes^14^. Additionally, lncRNAs might function as competing endogenous RNAs (ceRNAs) of miRNAs and may be involved in a range of cellular pathways. It has been known that large numbers of miRNA binding sites exist on a wide variety of RNA transcripts, including lncRNAs, leading to the hypothesis that lncRNAs contain miRNA-binding sites can communicate with and regulate the target mRNAs by competing specifically for shared miRNAs, thus acting as competing endogenous RNAs (ceRNAs) to protein coding mRNAs^15^. An example is the lncRNA cardiac hypertrophy related factor (CHRF) that directly regulates Myd88 expression as a ceRNA of miR-489, leading to cardiac hypertrophy^16^. HOTAIR and its targeted miRNA miR-34a also functioned in the process of prostate cancer cell growth inhibited by genistein^17^. This evidence indicated there was crosstalk between lncRNAs and small non-coding RNAs in disease development.

Endometriosis, a common estrogen-dependent gynecology disorder, affects 6 to 10% women of reproductive age, 50 to 60% of women and teenage girls with pelvic pain, and up to 50% of women with infertility. The disease is characterized by the presence of endometrium-like tissues outside the uterus, primarily on the pelvic peritoneum and ovaries^18^. This disease is diagnosed primarily by visualization during surgery, and the present gold standard for the diagnosis of endometriosis is surgical assessment by laparoscopy. As a result, diagnosis and intervention are often delayed due to the lack of sensitive biomarkers in the early stages of the disease^19^. Thus, biomarkers with high sensitivity, high specificity and low trauma for the diagnosis of endometriosis are needed. In addition, the pathogenesis of endometriosis is likely multifactorial, and several hypotheses have been suggested to explain the presence of ectopic endometrial tissue and stroma; these studies have provided novel biomarkers with potential use for the diagnosis of and treatment strategy for the disease^20^,^21^. However, it is clear that the pathways involved in endometriosis are complicated, and the molecular mechanisms that underlie the process are largely elusive.

In this study, we applied genome-wide profiling to investigate the tissue and serum lncRNAs that were differentially expressed between endometriosis patients and negative controls and to evaluate the potential of these lncRNAs as non-invasive diagnostic markers for the disease^22^. Furthermore, to better understand the potential roles of lncRNAs implicated in endometriosis progression, we further analyzed and predicted the functions of these dysregulated lncRNAs.

## Results

### Identification of differentially expressed lncRNAs between tissue and serum samples of endometriosis patients

**Figure 1:**
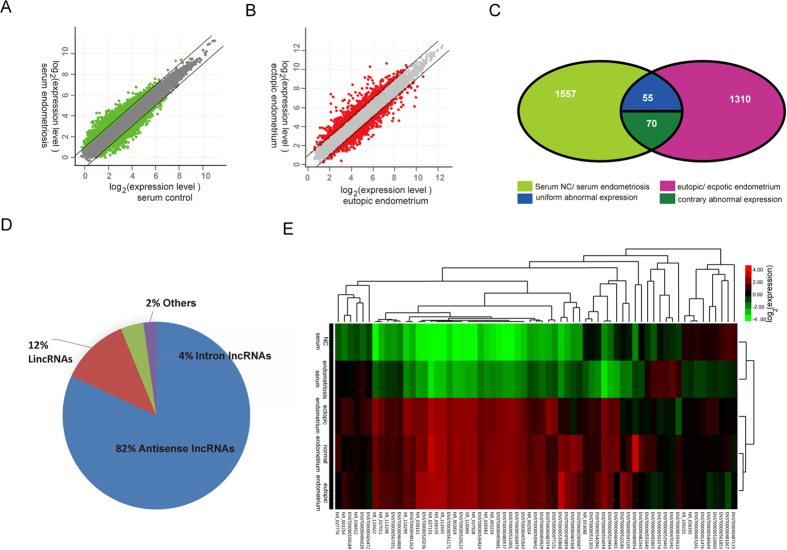
Abnormal expression of lncRNAs in serum and tissue from endometriosis and control patients. (**A**) Scatter plot of circulating lncRNA expression between the endometriosis and control samples; green spots show a difference >2. (**B**) Scatter plot of expression of lncRNAs between ectopic endometrium and eutopic endometrium; red spots show a difference >2. (**C**) Set diagram showing dysregulated lncRNA expression between the serum and tissue samples; (**D**) The deregulated lncRNAs were classified into different sets: retained introns (4%), lincRNAs (12%), or antisense RNAs (82%);(**E**) Cluster analysis of lncRNA expression in endometriosis patient serum and serum controls, eutopic endometrium tissue samples, ectopic endometrium tissue samples and negative tissue controls. The 65 top-ranked, differentially expressed lncRNAs are displayed (fold-change > 2.0). The expression values are represented in red and green, indicating expression above and below the median expression value across all samples, respectively. Each pooled sample has an array of data in the heatmap.

In an effort to identify lncRNAs that were differentially expressed between patients and negative controls, we first performed a genome-wide lncRNA expression study using the Glue Grant Human Transcriptome Array^23^, which contained approximately 39,223 lncRNAs. The arrays were performed with 5 sets of pooled samples, including a pool of 10 endometriosis serum samples, a pool of 10 control serum samples, a pool of 5 eutopic (EU) endometrium tissue samples, a pool of 5 ectopic (EC) endometrium tissue samples and a pool of 5 negative tissue controls. The array analysis identified 1682 lncRNAs with dysregulated expression (more than 2-fold change) in the sera of patients with endometriosis compared with controls (Fig. 1A) and 1435 lncRNAs in the ectopic endometrium compared with the eutopic endometrium (Fig. 1B). Furthermore, among the abnormally expressed lncRNAs, 125 lncRNAs were present in both the serum and tissue samples; 1557 lncRNAs were present only in the serum set; and 1310 were present only in the endometriosis tissue set (Fig. 1C). Additionally, among the 125 deregulated lncRNAs in both serum and tissue, 55 lncRNAs showed the same expression pattern (for example, ENST00000544649, ENST00000529000 and ENST00000481067 were up-regulated in both serum and tissue), while 70 of the 125 deregulated lncRNAs presented an opposing expression pattern. For example, the expression profiles of ENST00000426472, FR406817 and ENST00000477151 were increased in serum samples but decreased in tissue samples. With further re-analysis of the differentially expressed lncRNAs from the array, we classified the deregulated lncRNAs into different sets; i.e., retained introns, lincRNAs, or antisense RNAs. In the study, we found that antisense RNAs were predominated (~82%; Fig. 1D), which may be generally closed to host genes in the ensemble or NCBI database, indicating that they may present important roles in the process of the disease. Figure 1E shows the top 65 differentially expressed lncRNAs in the tissue and serum samples, which clustered into their own biological subtypes. The results suggested that the expression pattern and function of lncRNAs in serum may be different from that in tissue, which is similar to that of the miRNAs in the disease^24^,^25^,^26^. However, further studies are necessary to investigate the origin of circulating lncRNAs.

We next investigated the expression profile of lncRNAs among different tissue samples: EC, EU and negative endometrium controls. With unsupervised hierarchical clustering analysis, 60 lncRNAs had the most differential expression in these three types of tissue samples and clustered into their own biological subtypes (Figure S1).These differentially expressed lncRNAs may function in the development and processes of aeutopic endometrium, particularly those that are differentially expressed between the EU and control tissues, such as ENST00000393610, NR_033688, and ENST00000482343. Furthermore, we also analyzed the mRNA expression data in the comprehensive array and found large numbers of deregulated mRNAs (Figure S2), which may have the potential to serve as biomarkers for endometriosis^27^ and will also be important in our future studies.

### Validation of specific lncRNAs differentially expressed in serum and tissue and as potential diagnostic biomarkers for the disease

We next endeavored to further validate the lncRNA array accuracy and investigate the clinical application of serum lncRNA. Although relative quantification RT-PCR has been widely used for mRNA and small non-coding RNA detection, this method requires a suitable internal control, and no stable, suitable and recognizable standard internal controls have been used for lncRNAs in body fluids. Therefore, an absolute quantitation method was proposed for further validation. We therefore first initiated and developed a method for standard construction to quantify circulating lncRNAs. The lncRNA templates to construct standard curves were designed and synthesized; for detailed procedures, see the Materials and Methods section. The results showed the standard curve of these selected lncRNAs had good efficiency, R^2^ and slope^28^,^29^, indicating that the method was suitable for circulating lncRNA quantification (Figure S3).

**Figure 2:**
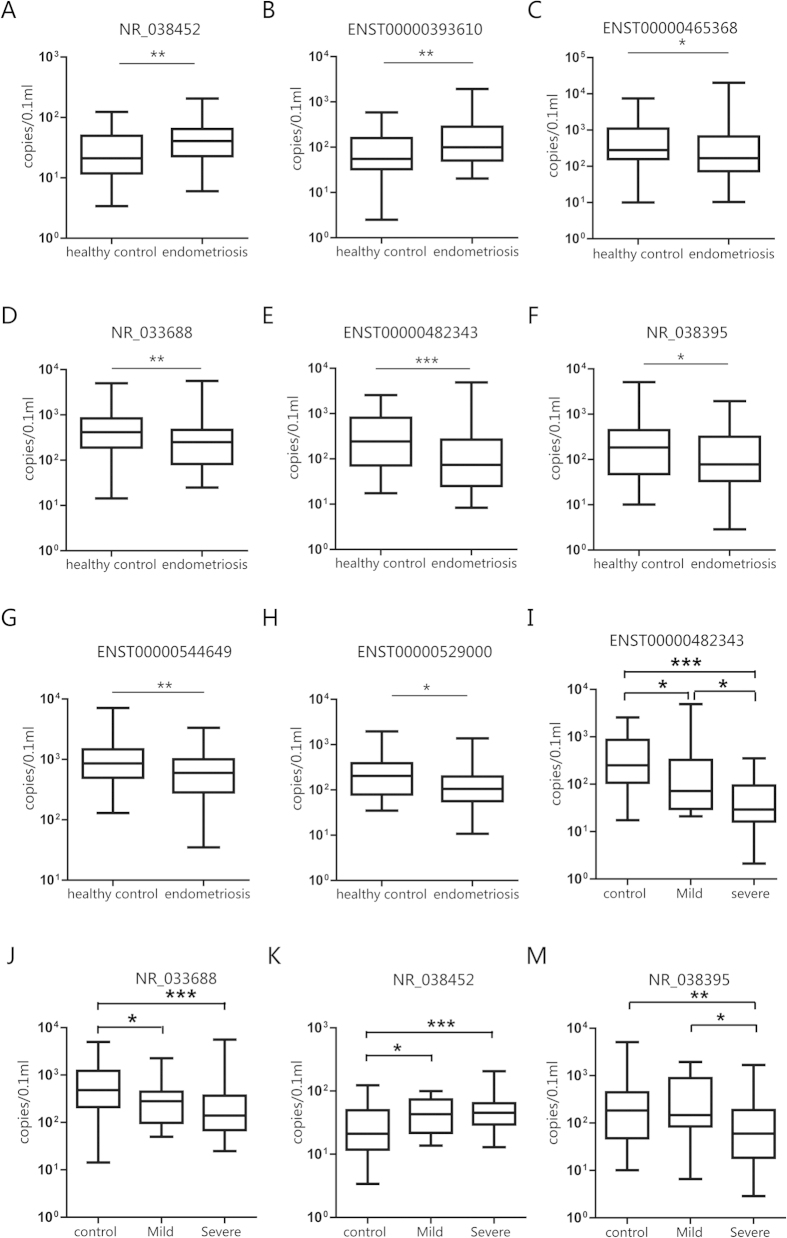
Circulating lncRNAs differentially expressed in patients with or without endometriosis. The expression levels of lncRNAs in serum samples from patients with endometriosis (n = 59) and control patients (n = 51) were detected with an absolute quantitative RT-PCR assay, and box plots illustrate the distinction. The copy numbers of NR_038452 (**A**) and ENST00000393610 (**B**) were higher in endometriosis patients than in the controls (P < 0.01), while the copy number of ENST00000465368 (**C**) P < 0.05), NR_033688 (**D**) P < 0.01), ENST00000482343 (**E**) P < 0.001), NR_038395 (**F**) P < 0.05), ENST00000544649 (**G**) P < 0.01) and ENST00000529000 (**H**) P < 0.05) were lower in endometriosis patientsthan in the controls. All P values were determined with a two-tailed Mann-Whitney U test. Additionally, altered expression levels of ENST00000482343 (**I**), NR_033688 (**J**), NR_038452 (**K**) and NR_038395 (**M**) were observed in patients with varying levels of endometriosis severity. Notably, the expression level of ENST00000482343 continued to decrease as the disease progressed (P < 0.05). A Kruskal-Wallis test was implemented among the 3 groups,and multiple comparisons were carried out using a *LSD-t* test. *, **, *** represents P < 0.05, P < 0.01, and P < 0.001, respectively.

**Figure 3:**
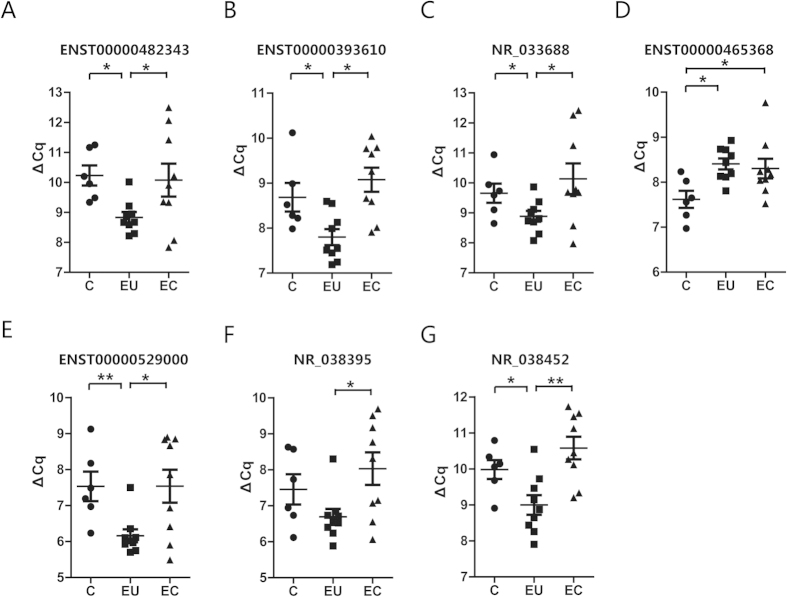
Aberrant expression profile of lncRNAs between the pairs of EU and EC endometriosis patient samples. The expression levels of special lncRNAs in the tissue samples, ENST00000482343 (**A**) ENST00000393610 (**B**) NR_033688 (**C**) ENST00000465368 (**D**) ENST00000529000 (**E**) NR_038395 (**F**) and NR_038452 (**G**). The pairs of EU and EC endometriosis patient samples (n = 9) and controls (n = 6) were accessed with a quantitative RT-PCR assay, and the resultsare shown by dot graphs A one-way ANOVA test was implemented when the comparison was among 3 groups of lncRNAs from tissue, and multiple comparisons were performed using a*LSD-t* test. *^,^ **^,^ *** represent P < 0.05, P < 0.01, and P < 0.001, respectively.

According to the method established, we chose 16 differentially expressed lncRNAs to validate the lncRNA array accuracy and investigate the clinical application of serum lncRNA. These selected lncRNAs presented significant deregulated expressions both in serum (serum NC/serum endometriosis group) and tissue (eutopic/ectopic endometrium group), and all of these lncRNAs presented with highly significant differences. Among those selected, 10 lncRNAs displayed the same expression patterns in both tissue and serum, and 6 lncRNAs showed the opposite expression patterns in serum and tissue. Using the absolute qPCR method and the standard curves specifically constructed for lncRNAs, we validated their expression in the serum sample set consisting of 59 endometriosis patients and 51 negative controls. Eight of the 16 selected lncRNAs could clearly distinguish the disease samples from the control group with high confidence (P < 0.05). For example, the expression levels of NR_038452 and ENST00000393610 were higher in endometriosis serum than in that of the controls, while the levels of ENST00000465368, NR_033688, ENST00000482343, NR_038395, ENST00000544649 and ENST00000529000 were lower in the disease patients than in the negative controls (Fig. 2A–H). We also investigated these particular serum lncRNAs in tissue samples, which consisted of9 paired EU and EC endometrium samples and 6 negative control endometrium samples (Fig. 3). In the tissue samples, except for ENST00000544649, 7 of the 8 lncRNAs were also found deregulated in endometriosis patients.

To explore whether these abnormally expressed lncRNAs could be useful for disease severity detection, we reanalyzed these lncRNAs in the subgroups of endometriosis patients serum samples at different stages, such as mild (stage I/II) and severe (stage III/IV). Figure 2I–M shows the four lncRNAs with different expression profiles in the subgroups. Notably, we found that the expression level of ENST00000482343 continued to decrease as the disease progressed (P < 0.05), whereas the expression levels of NR_033688, NR_038452 and NR_038395 consistently increased with disease severity, although no statistical significance was observed in this group. These results suggested that the expression levels of these lncRNAs may be associated with the severity of the disease.

### Investigation of circulating lncRNAs for use in the diagnosis of endometriosis

**Figure 4:**
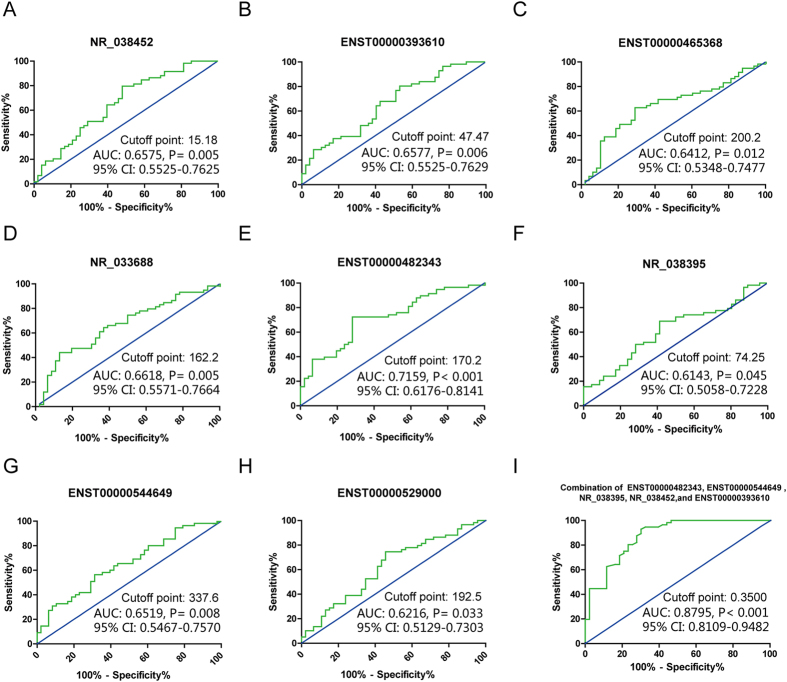
Assessment of the diagnostic accuracy of these special lncRNAs for endometriosis. Diagnostic value of serum lncRNAs for endometriosis: NR_038452 (**A**) ENST00000393610 (**B**) ENST00000465368 (**C**) NR_033688 (**D**) ENST00000482343 (**E**) NR_038395 (**F**) ENST00000544649 (**G**) and ENST00000529000 (**H**). The diagnostic power of the combination of the five specific lncRNAs (**I**): ENST00000482343, ENST00000393610, ENST00000544649, NR_038395, and NR_038452 for endometriosis.

The results described above showed that endometriosis patients display a highly characteristic lncRNA expression profile in both serum and tissue samples. We next endeavored to evaluate the diagnostic value of these aberrantly expressed lncRNAs for endometriosis. Receiver operating characteristic (ROC) curve analysis was performed for the expression of the lncRNAs mentioned above, and the associated area under the ROC curve (AUC), as well as the sensitivity and specificity, was used to confirm the diagnostic potency. As shown in Fig. 4, the highest AUC of a circulating lncRNA was for ENST00000482343, which reached 0.7159 [95% CI: 0.6176–0.8141, P < 0.001], with 72.41% sensitivity and 71.74% specificity at the cutoff point. We also found that NR_038395 had the greatest sensitivity, which was 84.75% at the cutoff point among the specific lncRNAs, whereas ENST00000544649 revealed the greatest specificity, which was 91.67% at the cutoff point. Previous studies have combined a single biomarker to improve the diagnostic power; therefore, we applied discriminant analysis to further investigate this possibility by analyzing multiple dysregulated lncRNAs. As a result, we achieved an optimal combination of NR_038395, NR_038452, ENST00000482343, ENST00000544649 and ENST00000393610 to differentiate patients with and without endometriosis. The following discriminant equation was determined: predicted value of probability (PVP) = 0.832*ln*ENST00000482343 + 0.230*ln*ENST00000544649-0.536 *ln*ENST00000393610-0.337*ln*NR_038395-0.124*ln*NR_038452-1.104. The AUC was as great as 0.8795 [95% CI: 0.8109–0.9482, P < 0.001], with 89.66% sensitivity and 73.17% specificity, at the cutoff point of 0.3500 (Fig. 4I). Following our careful assessment of the diagnostic value of the selected serum lncRNAs listed above, we suggest that specific circulating lncRNAs may have potential for detecting endometriosis.

**Figure 5:**
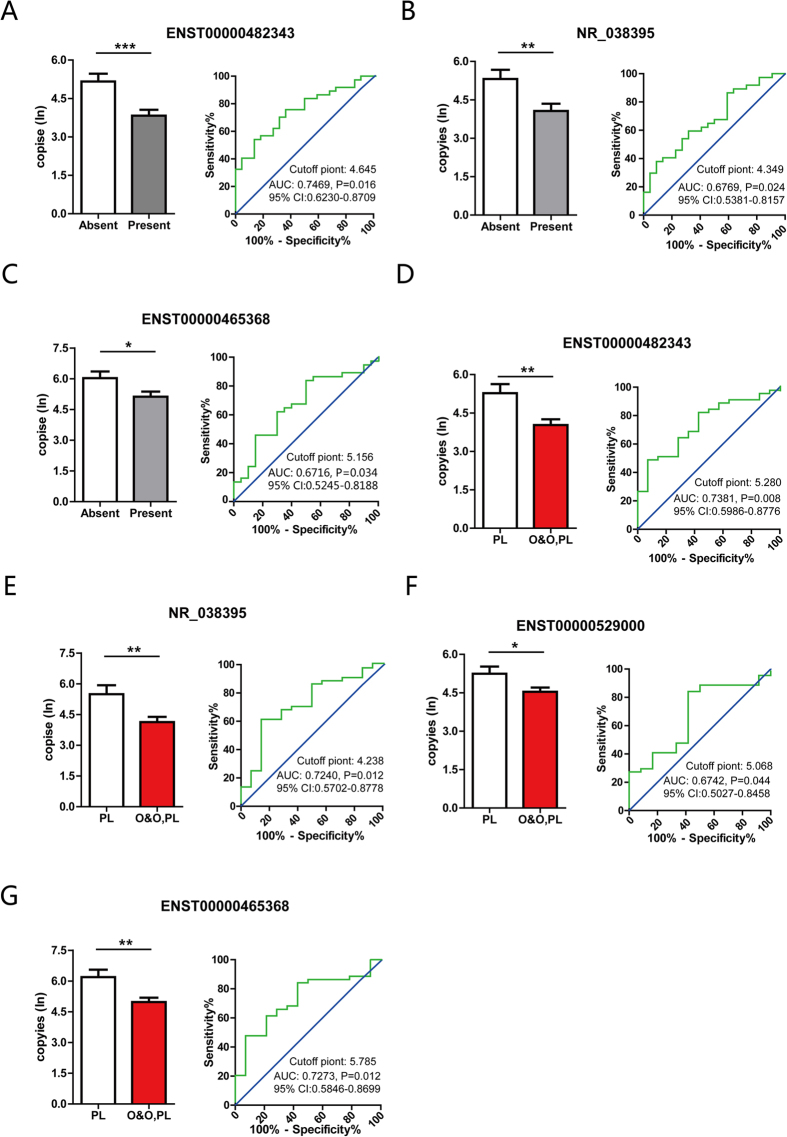
Association between lncRNA expression and clinical features of this disease. LncRNAs with different expression levels in serum from patients with different clinical features, such as with or without pelvic adhesion (**A–C**, left) and with or without ovarian involvement (**D–G**). (**A**–**G**), right, shows the diagnostic value of specific lncRNAs for the pelvic adhesion and ovarian involvement of endometriosis.

We also examined if the abnormally expressed lncRNAs were associated with the clinical features of this disease, including pelvic adhesion and endometriosis with ovarian involvement. Retrospective analysis of lncRNAs following Napierian logarithm transformation revealed that the expression levels of a set of lncRNAs varied; for example, ENST00000482343, NR_038395 and ENST00000465368 were decreased in sera from patients with pelvic adhesion caused by endometriosis (n = 37) compared with those without (n = 22; Fig. 5A–C). ROC curve analysis showed that ENST00000482343 presented the highest AUC of 0.7469 [95% CI: 0.6230–0.8709, P < 0.01] with 75.68% sensitivity and 63.64% specificity. Subsequently, a comparison of endometriosis with (n = 45) or without (n = 14) ovarian involvement indicated that ENST00000482343, NR_038395, ENST00000465368 and ENST00000529000 showed significantly decreased expression levels in patients with ovarian endometrioma (Fig. 5D–G). ENST00000482343 also had the greatest AUC of 0.7381 [95% CI: 0.5986–0.8776, P < 0.01], with 82.22% sensitivity and 57.14% specificity. Additionally, we correlated the expression of these circulatory lncRNAs with the menstrual cycle. However, only NR_038452 (P = 0.029) showed a difference in expression level between the follicular and luteal phases in patients with endometriosis. We also investigated other clinical features, such as infertility, and the degree of dysmenorrhea (mild, moderate, or severe); however, no statistically significant difference was found (data not shown). Together, these results suggested that lncRNAs may have the potential to detect endometriosis or distinguish the different pathological types of the disease.

### Differentially expressed lncRNAs might function in disease development through ceRNA crosstalk

We finally explored the biological process of deregulated lncRNAs, which may be considered potential markers for diagnostic endometriosis. Previous studies have hypothesized that numerous lncRNAs containing many miRNA binding sites can act as competing endogenous RNAs (ceRNA) that involve the posttranscriptional regulation of genes^15^,^30^,^31^; therefore, we constructed an lncRNA-miRNA crosstalk network using the Target Scan database, which can predict the direct interactions between miRNAs and lncRNAs^32^,^33^. As shown in Figure S4, a large number of lncRNA-miRNA pairs were predicted to have direct interactions. In this crosstalk network, an lncRNA that served as the ceRNA of a miRNA might have similar functions with the miRNA and its targeted genes; thus, their functional categories were analyzed with the Database for Annotation, Visualization and Integrated Discovery (DAVID)^34^.

**Figure 6:**
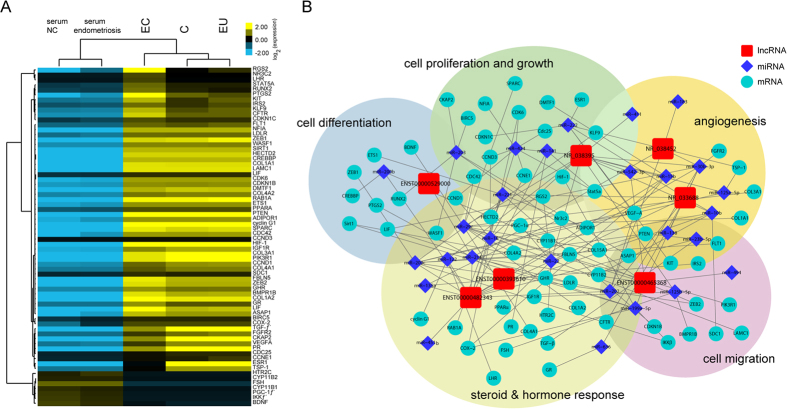
Graphical view of lncRNA-miRNA-mRNA network for lncRNAs. (**A**) Cluster analysis of the expression data of target genes using the GG-H array. Cluster analysis of lncRNA expression in endometriosis patient serum and serum controls, eutopic endometrium (EU) tissue sample, ectopic endometrium (EC) tissue sample and healthy tissue control (**C**). The 68 target genes are displayed. The expression values are represented in yellow and blue to show expression above and below the median expression value across all samples, respectively. (**B**) Graphical view of lncRNA-miRNA-mRNA network for 7 candidate lncRNAs. Boxes correspond to lncRNAs, diamonds correspond to miRNAs, circles correspond to mRNAs, and the edges correspond to direct interaction links. The most significant regions are marked with background colors, and the labels describe the main functions assigned.

We next constructed an lncRNA-miRNA-mRNA network using 7 lncRNAs deregulated in both serum (Fig. 2) and tissue samples (Fig. 3), together with 28 miRNAs and their target genes^23^,^26^,^35^,^36^ (Fig. 6A), which have been shown to play important roles in endometriosis in recent years. As shown in Fig. 6B, clustering of the lncRNAs, miRNAs and their target genes involved in several functional processes occurred, including the processes of cell proliferation and growth, cell differentiation and migration, and steroid or hormone responses, which are closely related to the development of endometriosis. For example, ENST00000465368 is predicted to act as a ceRNA of miR-199a, which targets and inhibits the IKKβ/nuclear factor-kappa B (NF-κB) pathway^35^, and suppresses proliferation, migration and angiogenesis of endometrial mesenchymal stem cells by targeting the VEGFA^26^, implying that the lncRNA may have the ability to enhance endometrial stromal cell invasiveness and contribute to the pathogenesis of endometriosis. Notably, a number of lncRNAs are predicted to be ceRNAs for many miRNAs; for instance, NR_033688 for miR-10b, miR-29c, and miR-200c. Further,miR-10b inhibits epithelial endometriotic cell invasiveness by targeting Syndecan-1 (SDC1)^36^, suggesting that NR_033688 may associate with disease migration, whereas, endometrial miR-200c influences many events during normal and disease progression, such as hormone response cellular transformation, inflammation, and angiogenesis, which indicates this lncRNA may be involved with hormone mediated endometriosis progression. These results showed that lncRNAs might be involved in different ceRNA crosstalk, contributing to the development of endometriosis. Further studies are necessary to confirm the crosstalk between lncRNAs and miRNAs in endometriosis pathogenesis.

## Discussion

Genome-wide human transcriptional studies have shown that large numbers of lncRNAs are deregulated in the disease process^1^,^2^,^37^,^38^. Many dysregulated lncRNAs have been identified in tissues or in body fluids, and these lncRNAs were reported to play important roles in disease development or act as non-invasive biomarkers^8^,^9^,^10^,^11^,^37^,^38^,^39^,^40^,^41^. However, studies on the identification and functional characterization of lncRNAs in gynecological diseases, especially in endometriosis, are limited. In this study, we investigated lncRNAs in the sera and tissues of endometriosis patients and identified a set of lncRNAs that can discriminate severe vs. mild stages of the disease and other associated clinical features. Furthermore, we attempted to characterize the function of dysregulated lncRNAs in endometriosis development through the ceRNA crosstalk network. This study is the first to report on circulating lncRNAs in gynecological disease, and it provides an understanding of lncRNAs that are associated with endometriosis.

In recent years, non-coding RNAs, such as miRNAs, have been employed as biomarkers with high sensitivity and specificity^24^,^46^,^47^ and also as the key regulators in cell processes^26^, suggesting that non-coding RNA molecules have potential roles in clinical diagnosis and in disease progression, for example, circulating miRNA let-7a–f and miR-135a,b for endometriosis^48^. Several studies have also reported that lncRNAs, which are similar in length to mRNAs, are stable in serum or body fluids and can not only enhance or inhibit disease development but also serve as potential biomarkers for many diseases^7^,^8^,^9^,^10^,^11^,^49^. For instance, Trimarchi *et al.* found a specific Notch-regulated lncRNA, LUNAR1, can enhance IGF1R mRNA expression andsustain IGF1 signaling in efficient T-ALL growth, and confirmed that lncRNAs are important regulators of the oncogenic state in T-ALL^13^.Serum lncRNA LIPCAR is considered a novel biomarker of cardiac remodeling and is predictive of mortality in heart failure patients^11^. In this study, we revealed that lncRNAs could serve as non-invasive biomarkers for endometriosis and may also contribute to the molecular pathogenesis of this disease.

LncRNAs are a class of molecules greater than 200 nt in length^1^,^2^, which might contain more genetic information than miRNAs, which are only 19~24 nt in length^50^,^51^. Because of this fact, circulating lncRNAs may present more information in serum when serving as non-invasive markers. We have found that the optimal combination of NR_038395, NR_038452, ENST00000482343, ENST00000544649, and ENST00000393610 can differentiate patients with and without endometriosis. These lncRNAs might have the potential for disease detection. In this study, we also found the expression levels of certain lncRNAs were related to the clinical features of this disease. Among these lncRNAs, ENST00000482343 was abnormally expressed in samples representing all of the clinical indicators, such as pelvic adhesion. A previous study has showed an association between lncRNA H19 expression during the menstrual cycle and the differentiation state of the human female reproductive tract^52^. In this study, we investigated the association of the differentially expressed circulatory lncRNAs with the menstrual cycle, however, only NR_038452 showed a difference in expression level between the follicular and luteal phases in patients with endometriosis. This may be the small sample sizes used or it may be the reason that the stages of the menstrual cycle may not affect the expression of most of lncRNAs in serum. Further study is necessary to validate the diagnostic value of circulating lncRNAs in a large cohort of samples.

Recently, both serum^5^,^6^,^24^ and plasma^8^ are used to extract circulating RNAs. In the plasma, there are different anticoagulants, such as EDTA, sodium oxalate, heparin, and trisodium citrate. Some of them, like EDTA, can affect the efficiency of PCR reaction^42^,^43^. So we used serum in our study and chose the Glue Grant Human Transcriptome Array microarrays for identification of the circulating lncRNA because the technique has been comprehensively designed to interrogate various aspects of the transcriptome, including gene expression, alternative splicing, and non-coding transcription. We also pooled samples to improve the products of circulating RNAs in serum. Previous reports have validated that pooled samples are good for circulating RNA research^44^,^45^. The disadvantage is that the expression pattern of circulating RNAs in each of sample cannot be obtained from the array data. Thus verification of the expression profile of circulating RNAs by qPCR in a set of samples is necessary. There is a challenge to quantify these molecules with a normal PCR method. One limitation of this approach is lack of stable, suitable and recognizable standard internal controls for lncRNAs. Thus, it is difficult to use relative quantification PCR to identify the differential expression patterns of circulating lncRNAs, and an absolute quantification method might be appropriate for lncRNA detection. Due to a shortage of synthetic analogues of lncRNA from commercial companies^53^, designing and obtaining a suitable lncRNA template for standard curve construction is essential. In this study, we have designed and cloned these selected circulating lncRNA templates, which were validated with good efficiency, R^2^ and slope. These synthetic analogues can be used to identify circulating lncRNAs and provide a source for novel lncRNA detection in body fluids.

It has been shown that the expression profile of lncRNAs presents spatial and temporal patterns^1^,^37^,^54^,^55^.Therefore, in this study, we examined the expression patterns of lncRNAs in normal, eutopic, and ectopic endometrium samples. A number of lncRNAs were differentially expressed in normal, eutopic, and ectopic endometrium samples, suggesting that they might function in the development and progression of endometriosis^56^,^57^. More importantly, we also found a number of lncRNAs that act as ceRNAs of miRNAs, and these lncRNAs were clustered according to many their biological processes, including cell proliferation and growth^26^,^36^, cell differentiation andmigration^26^,^35^, and steroid or hormone responses^36^,which are closely related to the development of endometriosis. For example, ENST00000465368 was suggested to have the related function of miR-199a, which has the ability to suppress the invasiveness, proliferation, migration and angiogenesis of endometrial mesenchymal stem cells^26^,^35^.NR_033688 may associate with disease migration for interaction with miR-10b, which inhibits epithelial endometriotic cell invasiveness by targeting Syndecan-1 (SDC1)^36^. These candidate lncRNAs that are aberrantly expressed both in serum and tissue and act as ceRNAs might provide new insight into the molecular mechanism of the disease. Further studies are necessary to validate the regulatory network between lncRNAs and miRNAs, as well as the target genes of the miRNAs associated with this disease.

In conclusion, we investigated the expression profile of lncRNAs in serum and tissue samples from patients with or without endometriosis. We also established a standard curve that had good efficiency for the quantification of circulating lncRNAs. Using the absolute qPCR method with the standard curves we obtained, we observed that the combination of five circulating lncRNAs, including NR_038395, NR_038452, ENST00000482343, ENST00000544649 and ENST00000393610, were potential non-invasive biomarkers for endometriosis. Our study also presented a possible candidate pool of lncRNAs in tissue for future functional studies associated with endometriosis. With the construction of a ceRNA crosstalk network, these candidate lncRNAs clustered in relation to various biological processes, suggesting that they may play important roles in the progression of endometriosis.

## Methods

### Patient and serum samples

The samples and clinicopathologic data were collected from the Department of Obstetrics and Gynecology, Sun Yat-sen Memorial Hospital (Guangzhou, China) in 2014. All the patients brought into this research were suffered from severe dysmenorrhea, pelvic mass or infertility. The negative controls were confirmed to be fallopian tubal diseases through laparoscopy and hysteroscopy, with neither endometriosis nor endometrial lesions. And for the positive cases, the inclusion criteria were as follows: 20–50 years old; no hormone therapy for at least 3 months; non-smoker; and no coexisting inflammatory disease. Women suffering from malignancy, benign ovarian cyst except endometrioma, severe pelvic inflammation observed during surgery, known chronic, systemic, metabolic, or endocrine disease including polycystic ovarian syndrome, were excluded from this study.

The study included 59 serum samples from patients diagnosed with peritoneal and/or ovarian endometriosis by laparoscopic and pathological examination and 51 control samples from patients primarily diagnosed with tubal factor infertilityand confirmed absence of endometriosis during their surgical procedure. Additionally, we examined 9 paired eutopic and ectopic endometrium samples from endometriosis patients and 6 negative endometrium controls from patients without endometriosis. The detailed clinical parameters of the cohort are presented in Table 1. Table S1 lists the pooled samples of the endometriosis and non-endometriosis patients in the array groups. No significant differences in age and BMI were found. Finally, all patients provided informed consent, and the study was approved by the ethics committee of Sun Yat-sen University. The sample collection and treatment were carried out in accordance with the approved guidelines.

### Serum and tissue processing and RNA isolation

The clinical blood samples from donors who fasted overnight were left for clotting at room temperature after collection and were then centrifuged within 1 h at 3000 rpm at 4 °C for 10 min to harvest the serum. Extraction of total RNA from 1 ml of the serum samples was achieved using the mirVana PARIS Kit (Ambion, TX). RNA was eluted with 100 μl of 95 °C pre-heated Elution Solution. Total RNA was isolated from tissue samples with TRizol (Invitrogen) according to the manufacturer’s instructions. The quantity and quality of total RNA was acceptable when there was an obvious absorbance peak at 260 nm, measured with a NanoDrop (Thermo Fisher, USA), and then, approximately 500 ng (~10 μg/μl) of RNA was obtained from 1 ml of serum. No difference in the amount of extracted RNA in a unit of serum was found between the control and endometriosis samples. The total RNA from tissue samples was used only if the ratio of the absorbance at 260 nm and 280 nm (A260/A280) was between 1.8 and 2.2^44^,^45^. All RNA samples were stored at 80 °C until further use.

For the Glue Grant Human Transcriptome Array, which included 39,223lncRNAs (Affymetrix, USA),circulating RNA was extracted from two pooled samples from 10 endometriosispatients or 10 non-endometriosis controls (each serum sample was 500 μl, and each pool contained 5 mL)^44^,^45^. The array data have been submitted to the NCBI GEO Archive (the accession number is GSE77182). Based on the results of lncRNA microarray analysis, specific primers for lncRNAs (Table S2) were synthetized and used to quantify lncRNAs in both serum and tissue samples. The reverse transcription of 2 μl of total RNA was carried out using a ReverTra Ace qPCR RT Kit (Toyobo, Japan). The levels of lncRNAs were measured in triplicate by SYBR Premix Ex Taq II-based (Takara, Japan) quantitative real-time PCR with ABI Stepone plus (ABI, American). The Cq value, which ranged from 15 to 35, was identified as applicable. Non-RT-PCR and no cDNA templates served as negative controls, and we therefore preformed an absolute quantitation method.

### Standard curve construction

Due to the current lack of stable, suitable and recognizable standard internal controls for lncRNAs in serum, we applied an absolute quantitation method^27^,^28^.In this study, we obtained the pure and accurate lncRNA standards in five steps. The first step was to harvest the target lncRNA template. The standards were analyzed in parallel with the clinical samples under identical qPCR conditions to calculate the start copies of clinical samples in a 20 μl SYBR reaction system. Reverse transcription was carried out using total RNA templates from a common cell line with specific primers. Target lncRNA sequences with ideal melt curves and sizes were identified using SYBR qPCR and 2.0% agarose gel electrophoresis. Second, to sequence the lncRNA templates, the target lncRNAs sequences were cloned and transformed into competent *E. coli* and were subsequently sequenced by Life Technologies (Thermo Fisher, USA), and the sequences that were 100% aligned in BLAST (Basic Local Alignment Search Tool) were considered acceptable. Bacteria carrying the desired sequences were cultivated, and the plasmids were extracted using the Plasmid Plus Midi Kit (QIAGEN, Genman). Third, to purify and retrieve the standards. Extracted by the AxyPrep DNA Gel Extraction Kit (Axygen, USA), standard samples were obtained following PCR using plasmid templates and 2.0% agarose gel electrophoresis. Standards were purified by 3 M sodium acetate and alcohol (overnight), and the A260/280 ratio of absorbance was in the range of 1.8–2.0, and the A260/230 ranged between 2.0 to 2.2. Fourth, to ensure exactness of the standards, the amplification efficiency, R^2^ and the slope^27^,^28^ were used to evaluate the standard curves of these lncRNAs. The results showed the standard curve of these selected lncRNAs had good efficiency, R^2^ and slope, suggesting the method was suitable for circulating lncRNA quantification. The expression levels of lncRNAs in serum were quantified by establishing standard curves with a set of serially diluted standard samples, the starting concentration of which was determined by spectrophotometry. Finally, we validated the standard curves within the circulating RNA samples, and almost all detectable signals of serum samples were on the standard curves, indicating that all of the standards were correct. We ensured the standards were exact and the method was reliable using the abovementioned methods.

### Statistical analysis

All statistical calculations and figures were performed using SPSS PASW Statistics (version 17.0) and GraphPad Prism (version 5.0). The GraphPad analysis was not only used for figure generations but also used to perform a Fisher’s exact test and Mann-Whitney U test, which were used to determine the significance of differentially expressed circulating lncRNA levels between the two groups. The Kruskal-Wallis test and a one-way ANOVA were used when the comparison was made among 3 groups of lncRNAs from serum and tissue, and multiple comparisons were made with aLSD-t test. Additionally, a parametric test was used with one-way ANOVA tests when the comparison was made among 3 groups for lncRNAs from tissue. There weretwo reasons for using non-parametric tests to address the circulating lncRNAs data. On the one hand, the inherent variation wasmuch greater, which didnot meet the condition for aparametric test; on theother hand, there was no statistical significance (P < 0.05) when the parametric test was used, such as anunpaired t test. SPSS PASW Statistics was used for ROC curve analysis, Youden’s index and discriminant analysis.The source code of the TargetScan database, which was used to search for the candidate targets of conserved 8mer and 7mer sites that matched the seed region of miRNA^32^,^33^ was used for searching the lncRNA that served as the ceRNA of miRNA. The DAVID^34^ was used to construct the gene network. All P values were two-tailed, and a P < 0.05 was considered statistically significant. All of the data were analyzed following Napierian logarithm transformation, and unpaired t tests and ROC curves were performed to determine the diagnostic utility of serum lncRNAs. The optimal cutoff point was chosen as the point at which Youden’s index was maximal.

## Additional Information

**How to cite this article**: Wang, W.-T. *et al.* Genome-wide Long Non-coding RNA Analysis Identified Circulating LncRNAs as Novel Non-invasive Diagnostic Biomarkers for Gynecological Disease. *Sci. Rep.* **6**, 23343; doi: 10.1038/srep23343 (2016).

## Supplementary Material

Supplementary Information

## Figures and Tables

**Table 1:** Clinical characteristics of all samples used in the study.

		Endometriosis (n = 59)	Normal control (n = 51)
**Age, mean ± SD**		32.34 ± 7.277	29.56 ± 4.841
**Dysmenorrhea**		31	22
**Main Diagnosis (Besides Endometriosis)**		Leiomyoma and Adenomyosis	Fallopian Tube Disease
**Stage of the menstrual cycle**			
	Follicular phase	50	44
	Luteal phase	9	7
**Pelvic adhesion**		Caused by endometriosis	Caused by inflammation
	Present	37	24
	Absent	22	27
**r-AFS Stage**			
	Stage I	12	NA
	Stage II	2	NA
	Stage III	30	NA
	Stage IV	15	NA
**Distribution of Endometriosis**			
	Ovarian Endometrioma	45	NA
	Peritoneal Lesion	14	NA
**DIE status**			
	With DIE lesions	4	NA
	Without DIE lesions	55	NA

NA, not applicable; DIE, deep infiltrating endometriosis; r-AFS, revised American Fertility Society.
