# Supplementary material for: Genome-wide Long Non-coding RNA Analysis Identified Circulating LncRNAs as Novel Non-invasive Diagnostic Biomarkers for Gynecological Disease
Source: Sci Rep. 2016 Mar 18;6:23343. doi: 10.1038/srep23343 (PMC4796908; doi:10.1038/srep23343)
Supplement: Supplementary Information [file srep23343-s1.pdf]

Supplementary Information

Title: Genome-wide Long Non-coding RNA Analysis Identified Circulating LncRNAs as Novel Non-invasive Diagnostic Biomarkers for Gynecological Disease

Running Title: Long noncoding RNAs in a Gynecological Disease

Wen-Tao Wang[1†], Yu-MengSun[1†], Wei Huang[1], Bo He[2], Ya-Nan Zhao[2], Yue-Qin Chen[1*]

†WTW and YMS contributed equally to this work.

1. Key Laboratory of Gene Engineering of the Ministry of Education, State Key Laboratory for Biocontrol, School of Life Science, Sun Yat-sen University, Guangzhou 510275, China.
2. Dept of Obst & Gyn, Sun Yat-sen Memorial Hospital, Sun Yat-sen University, Guangzhou 510120, China.

*Corresponding author:

School of Life Science, Sun Yat-sen University, Guangzhou 510275, P. R. China

Phone: 86-20-84112739；Fax: 86-20-84112399

E-mail: lsscyq@mail.sysu.edu.cn to Y.-Q. Chen

Table S1. Clinical characteristics of all pooled samples used in the GG-H array.

Serum samples (20)		Endometriosis(n=10)	Normal control(n=10)
**Age, mean±SD**		33.10 ±9.171	31.70 ±6.308
**Dysmenorrhea**		7	4
**Main Diagnosis (BesidesEndometriosis)**		Leiomyoma and Adenomyosis	Fallopian Tube Disease
**Stage of the menstrual cycle**			
	Follicular phase	8	8
	Luteal phase	2	2
**Pelvic adhesion**		Caused by endometriosis	Cuased by inflammation
	Present	9	8
	Absent	1	2
**r-AFS Stage**			
	Stage Ⅰ	1	NA
	Stage Ⅱ	1	NA
	Stage Ⅲ	3	NA
	Stage Ⅳ	5	NA
**Distribution of Endometriosis**			
	Ovarian Endometrioma	8	NA
	Peritoneal Lesion	2	NA
**DIE status**			
	With DIE lesions	2	NA
	Without DIE lesions	8	NA
**Tissue samples (15)**		**Eutopic endometrium and ectopic endometrium paired (n=5)**	**Normal control(n=5)**
**Age, mean±SD**		29.40 ±6.025	31.40 ±5.320
**Dysmenorrhea**		2	1
**Main Diagnosis (Besides Endometriosis)**		Leiomyoma and Adenomyosis	Fallopian Tube Disease
**Stage of the menstrual cycle**			
	Follicular phase	5	4
	Luteal phase	0	1
**Pelvic adhesion**		Caused by endometriosis	Cuased by inflammation
	Present	3	3
	Absent	2	2
**r-AFS Stage**			
	Stage Ⅰ	1	NA
	Stage Ⅱ	1	NA
	Stage Ⅲ	2	NA
	Stage Ⅳ	1	NA
**Distribution of Endometriosis**			
	Ovarian Endometrioma	3	NA
	Peritoneal Lesion	2	NA
**DIE status**			
	With DIE lesions	0	NA
	Without DIE lesions	5	NA

NA, not applicable; DIE, deep infiltrating endometriosis; r-AFS, revised American FertilitySociety

Table S2. Primers of lncRNAs in this study.

lncRNAs	Primer Forward	Primer Reverse	Length of template(bp)
**ENST00000482343**	AGGAGGTGCGGGAAAGTG	GGAGGAGGCAAAGAGGAACA	150
**NR_038452**	CCTTATGTGCCAGGAACTACTATCT	GGGAACTTGAAGGGTCATAGGT	151
**NR_033688**	TTCTGGTTCCGCCGATCTC	TGACTCTGATGCAGTGTGGG	159
**ENST00000529000**	CGGCTCATCACTGATTCCTAC	TCCGTGTCCACTCCTCCTG	125
**ENST00000544649**	ACCGTGACCACTCTTCTTCC	GAATCATCCACAAGGGCAAT	211
**ENST00000393610**	GTGGCACCAAGAGTGACCTG	CCAAGGCAGTCCCTATTTTCT	159
**ENST00000465368**	TGCCTCTTTAGCAATCTCAG	CTGCCTTTCTCACTTATGGTCT	108
**NR_038395**	TGACAAGCAAGACCCAAGGA	CAGAGTTAGGCAGGCATTTCA	126

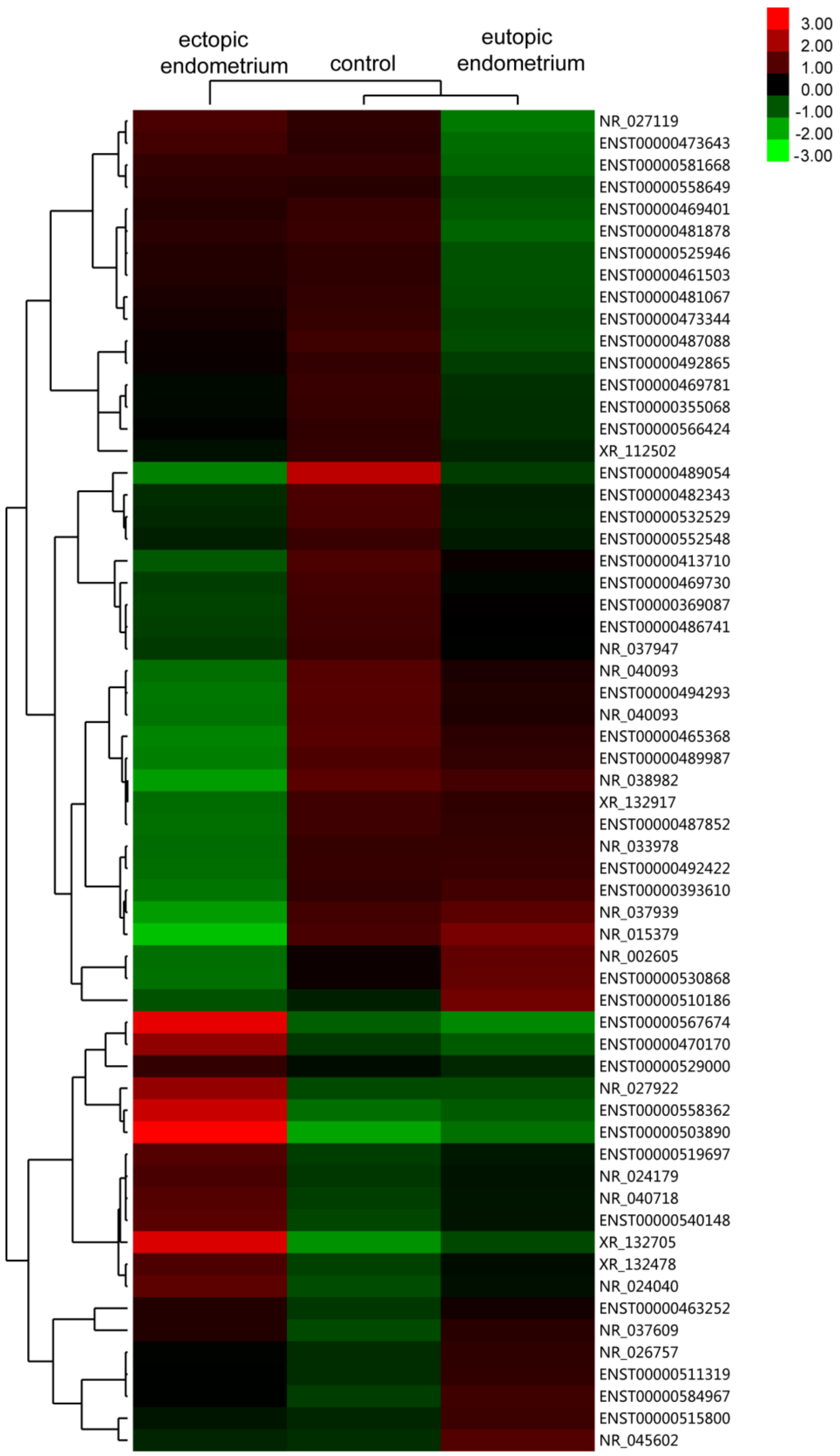

Figure S1. Cluster analysis of lncRNA expression in paired eutopic/ectopic endometrium tissue sample and negative tissue control. The 60 top-ranked differentially expressed lncRNAs are displayed (fold-change>2.0). The expression values are represented in red and green, indicating expression above and below the median expression value across all samples, respectively. Each pooled sample has an array data in the heatmap.

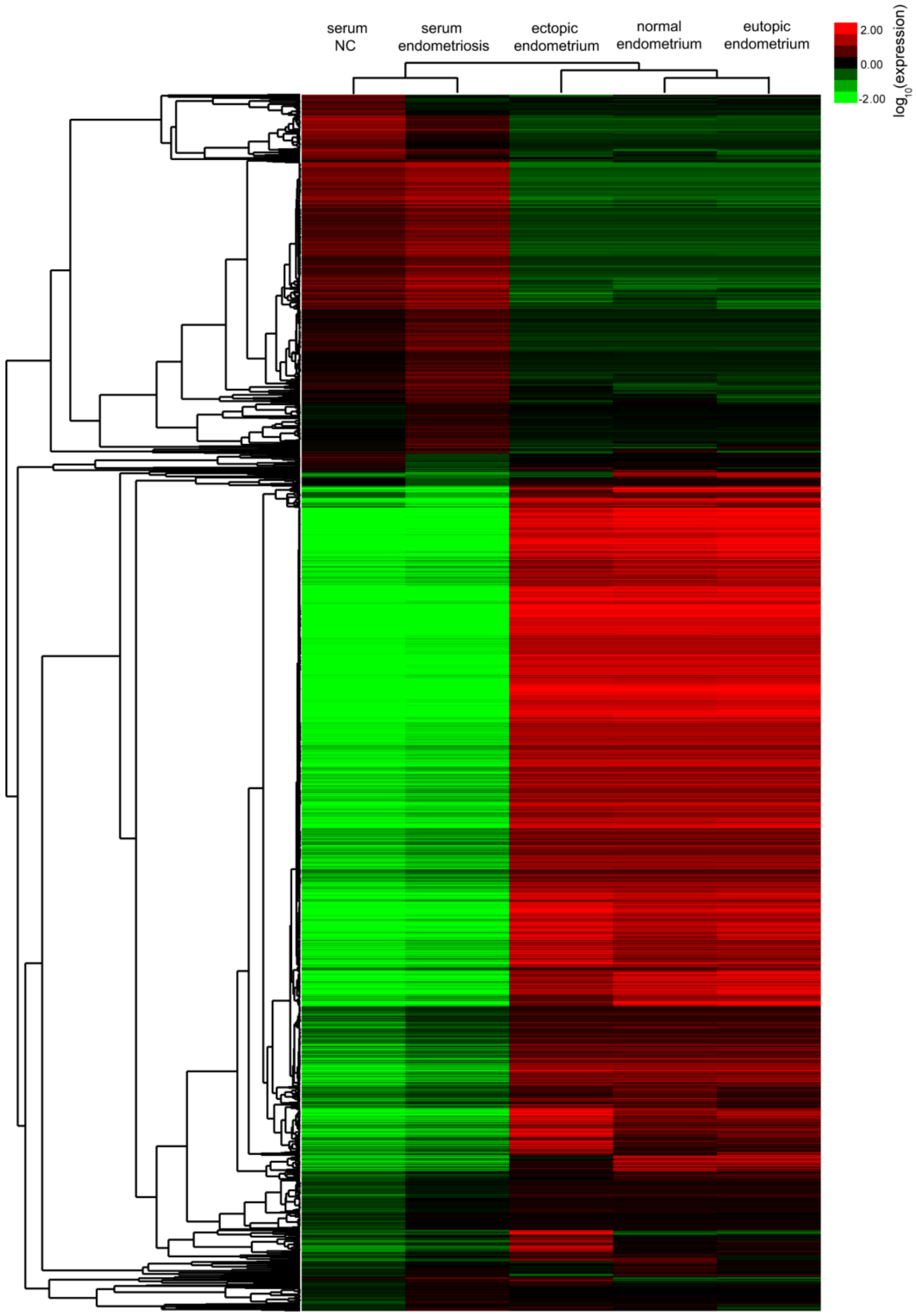

Figure S2, Cluster analysis of mRNA expression in serum and tissue samples. Cluster analysis of mRNA expression in endometriosis patient serum and serum controls, eutopic endometrium tissue sample, ectopic endometrium tissue sample and healthy tissue control. The differentially expressed mRNAs are displayed (fold-change>2.0). The expression values are represented in red and green, indicating expression above and below the median expression value across all samples, respectively. Each pooled sample has an array data in the heatmap.

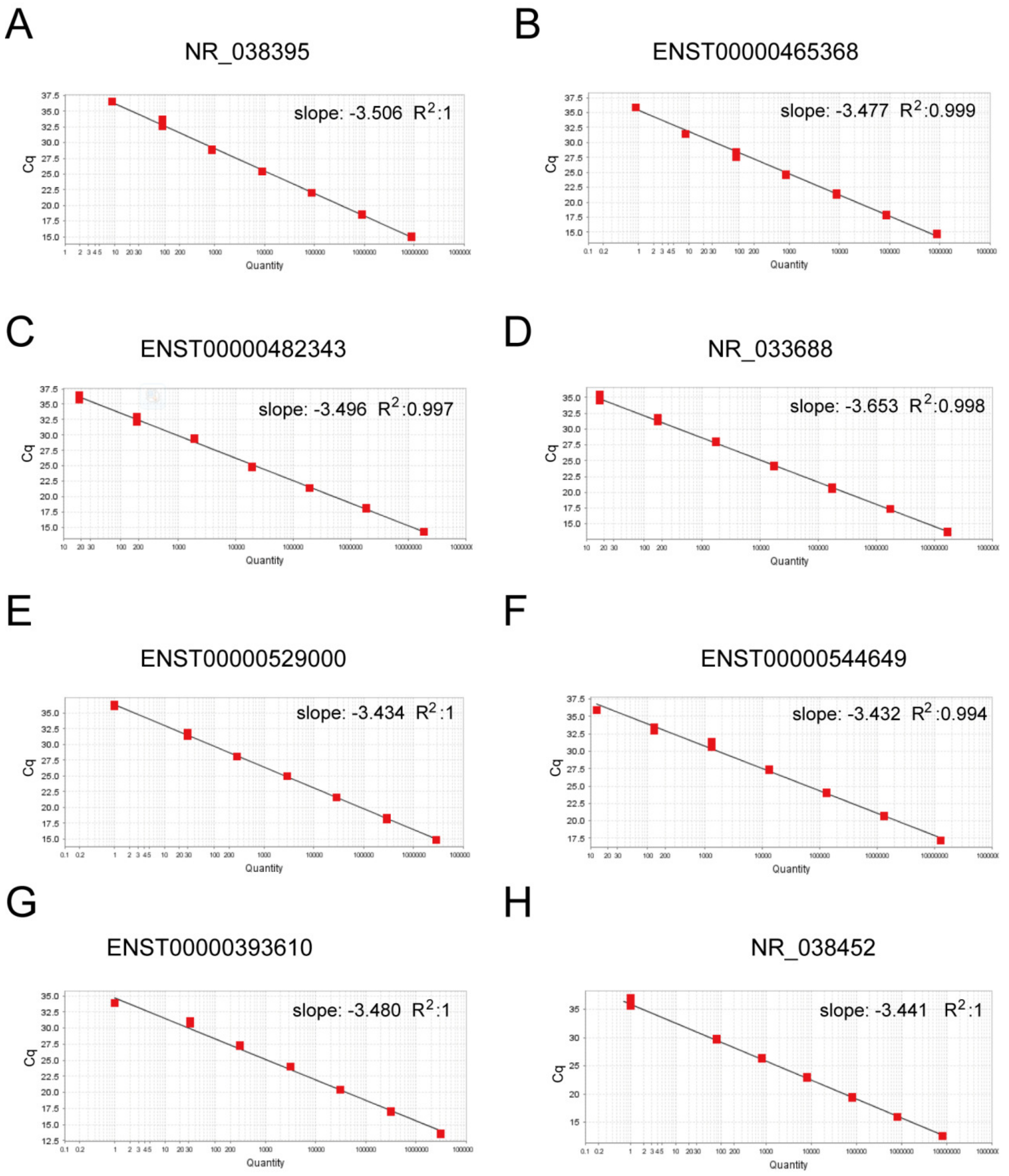

Figure S3. Standard curve construction for theseaberrantly expressedlncRNAs. The standard curve for those special circulating lncRNAs:NR_038395 (A), ENST00000465368 (B), ENST00000482343 (C), NR_033688 (D), ENST00000529000 (E), ENST00000544649 (F), ENST00000393610 (G) and NR_038452 (H).

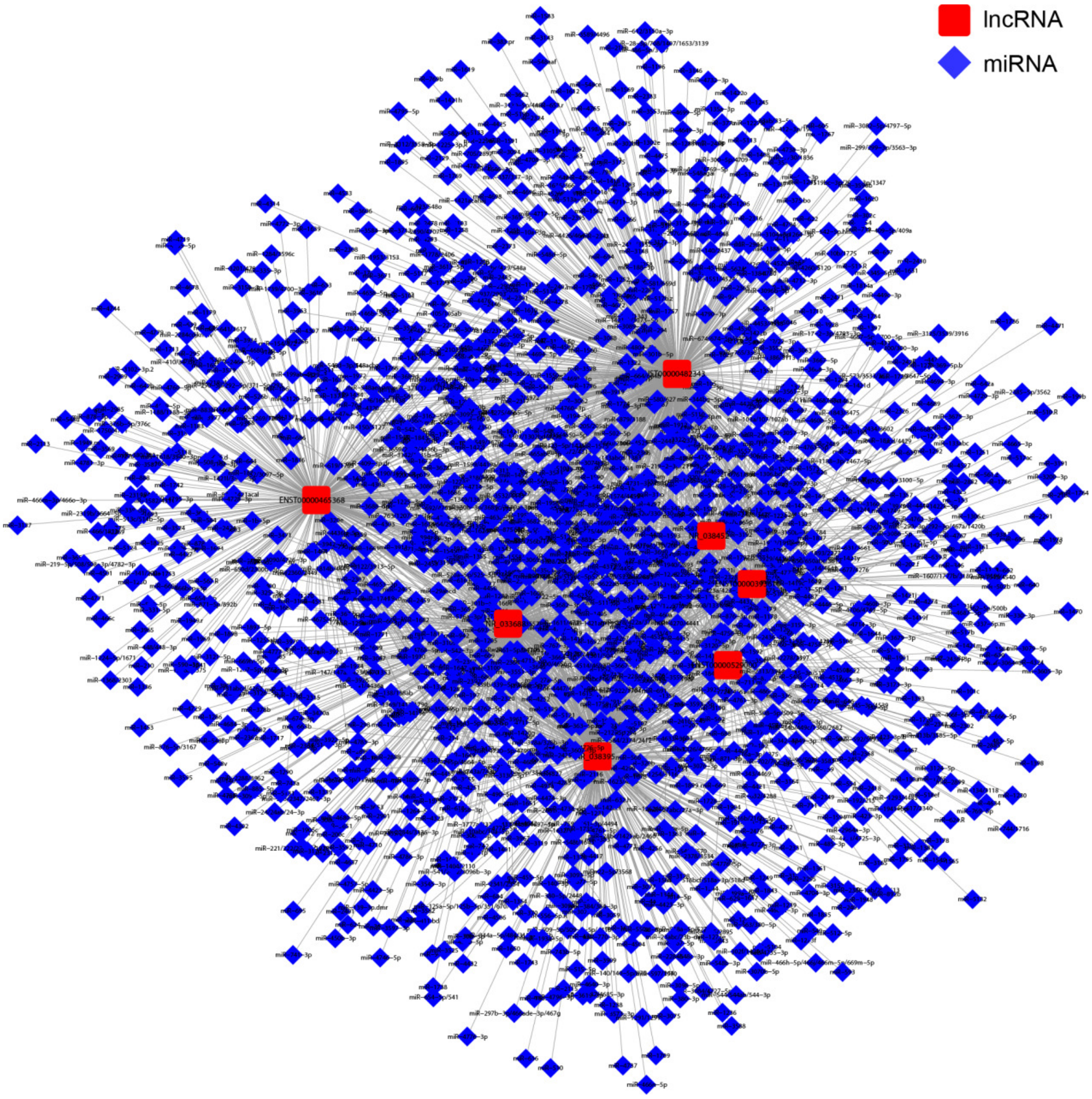

Figure S4. A global lncRNA-miRNA network for these special lncRNAs. Graphical view of the global lncRNA-miRNA network for 7 candidate lncRNAs. Boxes correspond to lncRNAs, diamonds correspond to miRNAs and the edges correspond to direct interaction links.
